# Supplementary material for: Patterns of Intron Gain and Loss in Fungi
Source: PLoS Biol. 2004 Nov 30;2(12):e422. doi: 10.1371/journal.pbio.0020422 (PMC532390; doi:10.1371/journal.pbio.0020422)
Supplement: Table S1 — Also available at http://genes.mit.edu/NielsenEtAl/. (4.3 MB ZIP). [file pbio.0020422.st001.zip › NielsenEtAl/html/259.html]

AN1363.1.NCU06735.1.MG02518.1.FG01545.1


```
 CLUSTAL W (1.82) Multiple Sequence Alignments - Introns Inserted


Sequence 1: NCU06735.1	682 aa
Sequence 2: MG02518.1	671 aa
Sequence 3: FG01545.1	523 aa
Sequence 4: AN1363.1	670 aa
Alignment Length: 687 aa
Number Identitical Residues: 299 aa
Alignment Score (without introns) 16031


MG02518.1 	-MADFDLGASFIPALHKPSSLLPIARHKDSLLYVVESNPVTVVVGQTGSGKTTQIPQFLE
NCU06735.1	MAADAGLDAGFIPALHKPSALLPIAKHREALLYTVETHPVTIVVGQTGSGKSTQIPQFLE
FG01545.1 	------------------------------------------------------------
AN1363.1  	MASELDVGTSFIPSLYKPASLLPIARHKQSILYLVETYPVTIVVGQTGSGKTTQLPQYLD
          	  :. . .:.  .:  ..::  . :   .:     .: . :   ..:.:..::. ..  .

MG02518.1 	KAGWCSDGKLIGVTQ0PRRIAATTVALRVAEEYGSEVGKEVGYSIRFEDVTSEATRIKFL
NCU06735.1	KAGWCADGKVIAITQ0PRRVAASTVAIRVAEEFGCELGKEVGFSIRFEDVTSEATRIKFL
FG01545.1 	---------------~--------------------------------------------
AN1363.1  	QAGWCTEGKAIAVTQ0PRRVAATTVAARVAEEMRCKLGEDVGYSIRFEDRTSAMTRIKFL
          	.:. .:...  . :. .   :::: :   :..  .. ... . :   .. ::  :  .  

MG02518.1 	TDGLLIREALVDPLLSRYSIVMVDEAHERSVSTDILLGLLKKIRKKRPELRIIISSATLQ
NCU06735.1	TDGLLIREALVDPLLSRYSVIMIDEAHERSISSDILLGLLKKIRKKRPELRIIISSATLQ
FG01545.1 	---------------------MVDEAHERSISTDVLLGLLKKIRRKRLELRIIISSATLQ
AN1363.1  	TDGMLLREALVDPLLSRYSVIMVDEAHERSLSTDILLGILKKILKRRPELRIIVSSATLQ
          	:..    .:  ..  :  :  *:*******:*:*:***:**** ::* *****:******

MG02518.1 	AEQFLAFFSSTLGGSR-----QDREEPQGTIGTI~VSLEGRTYPIDILYLESPAEDYLQR
NCU06735.1	AEEFLRFFSDSTGEAKSADNVQSDEKQDAPVGAI~VSLEGRTYPIDILYLEKPAEDYLEK
FG01545.1 	AKEFLEFFTSSSDGQTNSK--NGDNEKKDEIGAI0S----------------PAENYVEK
AN1363.1  	AEDFLRYFAGDESQQG------PNGEVGGNTGKI~ISIEGRMYPVDTLFLESAAENYVER
          	*::** :*:.  .            :     * *  : ..   . .    ...**:*:::

MG02518.1 	AVSTVLDVHENEPDGDILVFLTGRDEIEAAVDAVSERTAHLTSQDRGLLPLPLFSGLSTE
NCU06735.1	AISTVFDIHTNEPKGDILVFLTGRDEIEKAVEAVSERSAQLPVGSEAILPLPLYAGLPTE
FG01545.1 	AIDVVFDIHTQEGEGDILVFLTGREEIDNAIQAVSERMMDLGSKYGPLMPLPLYAGLSTE
AN1363.1  	SVKTVFDIHLQEGEGDILIFLTGREEIETAIQLISERATTLHPKSQSLLPLPLYAGLTTD
          	::..*:*:* :* .****:*****:**: *:: :***   *      ::****::**.*:

MG02518.1 	QQMYIFEEAPANTRKVIFSTNIAEASVTISGIVYVVDSGFVKLRAYNPKTGIETLTAVPI
NCU06735.1	KQMYVFDETPANFRKVIFSTNIAEASVTIDGIVYVVDSGFVKLRAYNPQTGIETLTATPV
FG01545.1 	QQMYVFDKLSEGTRKVVFSTNIAEASVTIDGIVFVVDSGFVKLRAYDPRTGIESLTATPV
AN1363.1  	QQMYVFEPAPENTRKVVVSTNIAEASVTINGIVYVVDCGFAKLRAYDPSTGIETLTAVPI
          	:***:*:  . . ***:.***********.***:***.**.*****:* ****:***.*:

MG02518.1 	SKAAAAQRAGRAGRTKPGKCFRLYTEESYTSLSDTNIPEVQRSNLAPFILQLKALGIDNV
NCU06735.1	SKASAAQRAGRAGRTKAGKCFRLYTEEAYQALHDANPPEIQRSNLAPFVLQLKALGIDNV
FG01545.1 	SKAAASQRAGRAGRTKPGKCFRLYTEQSYQSLQDANIPELQRSNLAPVVLQLKALGIDNI
AN1363.1  	SKAAAVQRAGRAGRTKPGKCFRLYTQHAYEQLPDATIPEIQRSNLAPVIMQLKALGIDNI
          	***:* **********.********:.:*  * *:. **:*******.::*********:

MG02518.1 	LHFDFLTPPPAELMTKALELLYSLGALDDYAKLTKPLGLRMAELAVDPMMAKTLLSAESF
NCU06735.1	LRFDFLTPPPAELMTRALELLYSLGALDDYAKLTKPLGLRMAELAVEPMMAKTLLSAPSF
FG01545.1 	VRFDFLSPPPSELMAKALELLYALGALDEYAKLTRPMGSRMAELAVEPMMAKTLLAAPSF
AN1363.1  	VRFDFLTSPPADLVIRAFELLYSLGAIDDYAKLTKPLGMRMAELAVDPMMAKVLLSAQSF
          	::****:.**::*: :*:****:***:*:*****:*:* *******:*****.**:* **

MG02518.1 	GCLSEMLTIAAMTSAGGDVWIQHEGERKQFESRRRKFAAGEGDHLTFLNVYQAFVTKGRK
NCU06735.1	GCLGEMLTIAAMTSLGGSIWVQPDGGKDEAESARRKFAADEGDHLTLLNVYQAFVTKGRK
FG01545.1 	GCLSEVLTIAAMTSLGGSVWFSHEGERKKMETSRRKFAVEEGDHLTLLNAYQAFVTKGRK
AN1363.1  	GCLSEILTIAAMVSLQGAVWVQHDGDKKSSESSRRKFAVEEGDHLTYLNVYQAFVTKGKK
          	***.*:******.*  * :*.. :* :.. *: *****. ****** **.********:*

MG02518.1 	ESGFCHENLLNYKTMSRAVSIRAQLKRYLERFGIRVD----ETLASGKTRGASPGVT-GE
NCU06735.1	EARFCHDNLINFKAMTRAMSIRAQLRRWLERFGITEDGLSVPQPAAAAAAAAAAGVNKAE
FG01545.1 	EAKFCHENNLNYKSMSRAISIRAQLKRYLERFSINVD----ETLAG--QASSEDNAKKAE
AN1363.1  	ESKWCRDNLLNYRSLLRAVSVRAQLKRYLERFGIQVD----ETFPS--HYNKSDLSKPAE
          	*: :*::* :*:::: **:*:****:*:****.*  *       ..          . .*

MG02518.1 	QIRRCLTTGYFAHAARMQADGTFQTVQGGTTLHAHPSSLMF~NRKADWVIFHEVMETGAK
NCU06735.1	QIRRCLTTGYFAHAARMQPDGSFRNVSGTTVLHAHPSSLMF~NRKADWVIFHEIMESGEK
FG01545.1 	QIRRCLTSGYFAHAARMQPDGTFRNVEGNMVLHAHPTSLMF~NRKADWVIFHEAMETGSK
AN1363.1  	KIQRCLTTGYFAHAARMQPDGTFKTVSGGMTLHAHPSSLMF0NRKADWVIFHEIMQTGEK
          	:*:****:**********.**:*:.*.*  .*****:**** *********** *::* *

MG02518.1 	TFIRDISKVEKCWLLEYAPDFYQVK-----
NCU06735.1	TFIRDITKIEKNWLVEYASAFYSTM-----
FG01545.1 	IFIRDVTKIEKTWLLEYAPEFYQITTDRRG
AN1363.1  	TFIRDITKIEKSYLLEYAPNYYNMQ-----
          	 ****::*:** :*:***. :*.
```
